# Development and validation of the living with pulmonary hypertension questionnaire in pulmonary arterial hypertension patients

**DOI:** 10.1186/1477-7525-11-161

**Published:** 2013-10-03

**Authors:** Nicola Bonner, Linda Abetz, Juliette Meunier, Mirko Sikirica, Stephen C Mathai

**Affiliations:** 1 Adelphi Values, Adelphi Mill, Bollington, Macclesfield, Cheshire SK10 5JB, UK; 2 Mapi Consultancy, Mapi Consultancy, Le D'Aubigny, 27 rue de la Villette, 69003, Lyon, France; 3 Bayer Pharma AG, Muellerstr 178, Building 13353, Berlin, Germany; 4 Division of Pulmonary and Critical Care Medicine, Johns Hopkins University School of Medicine, Baltimore, Maryland, USA

**Keywords:** Pulmonary arterial hypertension, Face and content validation, Psychometric validation, Living with pulmonary hypertension questionnaire

## Abstract

**Background:**

The Living with Pulmonary Hypertension questionnaire (LPH) was adapted from the Minnesota Living with Heart Failure Questionnaire for use in patients with pulmonary arterial hypertension (PAH). Study objectives were to confirm the face and content validity, to assess the structure and psychometric properties, and provide guidance for the interpretation of the LPH.

**Methods:**

A qualitative interview study was conducted with PAH patients in the US (n=12), Germany (n=14) and France (n=12) to evaluate the face and content validity of the LPH. Psychometric validation was performed using blinded data from a double blind, Phase III, clinical trial (n=196). Validation analyses were performed on baseline and week 12 (visit 6/last visit) data and included evaluation of: item response distributions, quality of completion, construct validity, reliability, clinical validity and responsiveness. Analyses to provide an estimation of the Minimal Important Difference (MID) for the LPH scores were performed.

**Results:**

Cognitive debriefing interviews with 38 PAH patients indicated that the most commonly reported PAH symptoms and impacts are covered by LPH items. Patients found the LPH questionnaire relevant and comprehensive to their experience. Some suggestions were made to enhance the face validity of the LPH. The content validity of the questionnaire was supported. Results of the psychometric validation analyses (n=190) indicated that the LPH Emotional and Physical scores met the criteria for convergent and discriminant validity; for the total score all but two items met the test for item convergent validity. Internal consistency reliability was demonstrated by Cronbach’s alpha values of >0.70 for all LPH scores. The LPH Physical and Total scores discriminated between World Health Organisation (WHO) Functional classes and 6 Minute walk test distances, indicating clinical validity and were also responsive to change in clinical severity, as measured by change in WHO functional class and Borg CR 10 Scale. Further investigation is required to confirm the responsiveness of the Emotional score. Estimation of MID using distribution-based methods indicated a change of 3 points for the sub-scales and 7 for the total score to be clinically meaningful.

**Conclusion:**

The LPH is a valid and reliable instrument that meets FDA criteria.

## Background

### Pulmonary arterial hypertension

Pulmonary arterial hypertension (PAH) is a rare lung disorder with current estimates suggesting the prevalence of PAH in the US is 109 per million individuals [1]. In PAH, pulmonary vascular injury leads to vessel remodeling and subsequent narrowing of the pulmonary arterioles that in turn, increases afterload on the right ventricle. As this disease progresses and afterload increases, right ventricular failure ensues that ultimately leads to death [2]. While the initial symptoms of PAH are non-specific, including shortness of breath (dyspnea) [3] and fatigue following physical exertion, these often progress to occur with minimal exertion or, in extreme cases, at rest [4]. Additionally, patients often experience swelling in the ankles or legs (edema); bluish lips and skin (cyanosis); chest pain; and palpitations [5].

Such debilitating symptoms result in substantial impairments in patients’ Health-Related Quality of Life (HRQoL) [6]. In particular, PAH results in significant impairment in physical functioning, ability to perform activities of daily living and social functioning, and many patients experience feelings of depression and anxiety and difficulties sleeping [7-9]. To evaluate treatment benefit, primary and key secondary endpoints in PAH clinical trials are typically clinical measures focused on sub-maximal exercise capacity (e.g. the six minute walked distance [6MWD]). However, to complement such trial endpoints, rigorous measurement of HRQoL related to PAH is also recommended to ensure treatments and interventions improve not just objective functional capacity, but also the day-to-day well-being of patients. To date, HRQoL measurement in trials has typically relied on generic measures which may not fully evaluate the specific impacts experienced by PAH patients [10,11]. Few disease-specific measures of quality of life (QoL) in PAH exist and are limited by the lack of explicitly defined responsiveness and clinical utility [12]. Thus, we sought to develop and assess a PAH-specific tool to measure HRQoL in this patient population by modifying an existing disease-specific metric of QoL in left heart failure, the Minnesota Living with Heart Failure questionnaire (MLHF). Further, we sought to define a minimal important difference for this new tool, the Living with Pulmonary Hypertension Questionnaire (LPH).

## Methods

### Objectives

The objectives of this work were to select and adapt an existing disease- specific measure of HRQoL for use in PAH populations and then to confirm the face and content validity and scoring and psychometric properties of the selected instrument. Specific objectives of the face and content validity testing of the LPH questionnaire were: to explore the item coverage of key symptoms and impacts of PAH; to explore whether the items, instructions, recall period and response options are relevant, well-understood and interpreted in a consistent manner by patients with PAH; and to identify any changes to the wording of the LPH that are recommended as a result. Specific objectives of the assessment of the psychometric properties of the LPH were: to confirm the item-scale structure of the LPH; to assess the reliability, validity and responsiveness of the LPH; and to provide guidance on the interpretation of LPH scores and changes in scores.

### Development of a conceptual model

**Figure 1:**
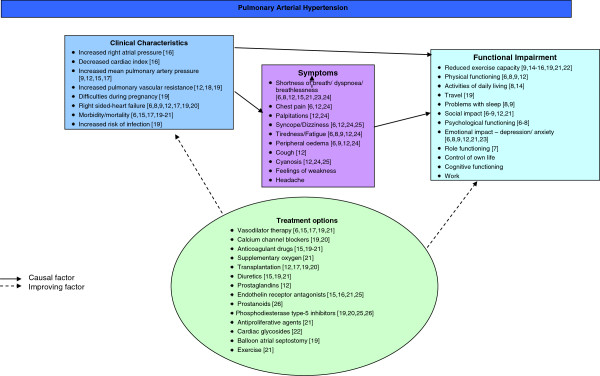
**PAH Conceptual Model**[6-9,12,14-26]**.**

Qualitative and quantitative PAH literature was reviewed to support the development of a conceptual model of PAH (Figure 1). The model includes a summary of clinical characteristics of PAH; symptoms experienced by patients and the resulting functional impairments, and available treatment options. The conceptual model was used as basis for a review of existing HRQoL instruments and to explore appropriate outcome measures to assess PAH. The instrument review identified only one PAH-specific instrument the Cambridge Pulmonary Hypertension Outcome Review (CAMPHOR) [12]. Although the CAMPHOR had been developed for specific use in PAH and there was evidence of the reliability and validity of the instrument [12] there were concerns about the concept coverage; there is no evidence that saturation analysis was conducted to confirm that all concepts important to patients were captured, mapping of the CAMPHOR concepts to the PAH conceptual model identified key concepts such as dizziness, chest pain and palpitations were missing from the CAMPHOR. There were also concerns about the length of the instrument (65 items) and the dichotomous format of some of the response options. Such factors would be a concern for regulatory agencies such as the FDA and may impact on the content validity and responsiveness of the instrument [13].

Given the concerns about the CAMPHOR, the MLHFQ [27] was identified as a stronger instrument, that provided better measurement and more comprehensive coverage of PAH symptoms and impacts. Moreover, there is evidence of previous use in PAH clinical trials [14,15,28,29], and that it is responsive to changes following treatment in PAH [15,29-31]. The MLHFQ however, is specific to heart failure. In order to make the instrument appropriate for use in patients with PAH, minor modifications were made to the MLHFQ. Modifications included: changes to the wording of some of the questions and instructions to be specific to PAH rather than heart failure, and a revision to the recall period from four weeks to one week. The reduction in the recall period to one week was considered an important modification in order to ensure the instrument met the FDA preference for “short recall periods” [13]. This work was conducted in order to provide a suitable instrument for the assessment of PAH symptoms in a clinical trial to benefit PAH patients across the globe.

### Qualitative patient interviews

After obtaining the relevant institutional review board approval (approval codes: 2009-P-001852/2, MAPI-10-242, B-F-2010-033, 09/2295), qualitative patient interviews were conducted to evaluate the content validity of the LPH. The qualitative interviews were conducted in the US, France and Germany and included patients with PAH as defined by current consensus guidelines [32] who were at least 18 years of age and had provided written informed consent. The MLHFQ had previously been translated and linguistically validated for use in France and Germany. Patients were either treatment naïve or had previously received treatment with an endothelin receptor antagonist (ERA), a phosphodiesterase type 5 inhibitor (PDE5I), or a prostacyclin analogue. Patients were required to have cognitive and linguistic capacities sufficient to allow them to actively participate in an interview, as determined by the recruiting physician. Patients with significant psychiatric disease were excluded from the study. Patients with a diagnosis of other relevant pulmonary diseases including pulmonary hypertension other than PAH, moderate to severe obstructive lung disease, or severe restrictive lung disease were excluded from the study [32].

Interviewers trained in qualitative research, native to each country, followed a semi-structured interview guide. The guide included both open-ended questions about patient experiences of PAH and a cognitive debriefing exercise to assess the patient’s understanding of the instructions, items, response options and recall period of the LPH. Examples of open-ended questions exploring the patient experience of PAH included:

“What is it like to have PAH?”

“Please tell me about any symptoms or problems that you experience, if any? When do you usually experience these symptoms? How long do these symptoms normally last for? In what ways do these symptoms affect you, if at all?”

“Does your PAH ever stop you from doing things? (e.g. housework, going out, hobbies) If yes please explain”

The cognitive debriefing part of the interview asked the patients general questions about the LPH questionnaire such as:

“What is your overall opinion of this questionnaire? Where there any questions that were unclear or difficult to answer? If yes, which? What made this/these questions difficult to answer?”

“What did you think about having to think about your symptoms over the past week?”

“Do you think it was relevant to think about your symptoms over the past week?”

Patients were also asked specific questions about each item or instruction, for example:

“What did you think of this question? What did this question mean to you, in your own words?”

“Was there anything that was unclear or hard to understand in this question? How would you reword it to make it clearer”

“How far back were you thinking when you answered this question? How long do you thinking this question was asking you to think back over?”

“Do you think the response options for this question are appropriate? Would you change them in any way?”

Procedures were implemented to capture any adverse events reported during the interviews; no adverse events were reported. Qualitative analysis of verbatim transcripts was performed using Atlas Ti software and Microsoft Excel and methods derived from Grounded Theory [33]. French and German transcripts were translated into English prior to analysis. To determine whether all of the symptoms and impacts of importance to patients with PAH had been elicited during the interviews, the patient interview sampling strategy and analysis followed the principle of 'saturation’. Saturation is defined as the point where no 'new’ information on a particular item or topic is mentioned by patients [34]. Interviews were analyzed in a stepwise manner to determine the point at which saturation was reached.

### Psychometric validation study

Psychometric validation of the LPH was performed using blinded data from a double blind, Phase III clinical trial. As part of this trial patients were randomized at Visit 1 (V1), and were then followed for 12 weeks. The LPH was self-administered at V0 (baseline) and V6 (12 weeks or last visit), the V6 time point was used for cross-sectional analyses. Patients with PAH, defined as per consensus guidelines, aged between 18 and 75 years of age, whose six-minute walk distance (6MWD) was between 150 and 450m were enrolled [32]. Patients were either treatment naïve or had previously received treatment with an ERA or a prostacyclin analogue. Patients were excluded from the trial if they were unable to perform the six-minute walk test (6MWT), had taken intravenous (IV) prostacyclin analogues, or PDE5I within the 90 days prior to visit 1.

### Measures

#### LPH Questionnaire

The LPH derived from the MLHF questionnaire comprises 21 items, responded to on a 6-point Likert scale ranging from 0 'No’ to 5 ’Very much’. A total score ranging from 0 to 105 is calculated by summing the responses to all 21 questions. A physical dimension score (range 0–40, 8 items) and an emotional dimension score (range 0–25, 5 items) can also be calculated. For all LPH scores, a higher score indicates that patients are more affected by their medical condition. The MLHFQ from which the LPH was derived has shown to be highly reliable as demonstrated by the correlation between repeated baseline assessments (r=0.93) [27]. The MLHFQ has high internal consistency validity, and an analysis of MLHFQ scores according to New York Health Association (NYHA) functional class supported the clinical validity of the MLHFQ [27]. It should be noted that these properties are relevant to patients with heart failure, not PAH. The MLHFQ has been used previously in PAH clinical trials. Cenedese et al. used the MLHFQ in a study of patients with PAH and chronic thromboembolic pulmonary hypertension (CTEPH). This study supported the internal consistency and test-retest reliability of the instrument in PAH and CTEPH patients as well as the clinical and concurrent validity and responsiveness [30]. In addition to the study by Cenedese et al [30] the MLHFQ has previously been used in other PAH clinical trials, where it has shown evidence of responsiveness to change following treatment [15,29,31].

### 6MWT

The 6MWT was conducted according to American Thoracic Society guidelines [35]. Specifically, the test was performed indoors, along a long, flat, straight, enclosed corridor of at least 30 meters in length. The 6MWT was conducted unencouraged by a person not involved in the titration of the study drug, who was unaware of the immediate reaction of the patient’s blood pressure and heart rate after dosing.

### The Borg CR10 scale

The Borg Category Ratio 10 (CR10) Scale was measured in conjunction with the 6MWD Test during the clinical trial. Patients are asked to rank their exertion at the end of the 6MWD test on a scale with the lowest rating being '0 Nothing at all’ up to the patients being able to rate their exertion as a ’12 or still higher’ which represents “Absolute maximum”. The Borg CR 10 Scale has been shown to be a valid and reliable measure for the estimation of perceived intensity [36,37].

### World Health Organisation (WHO) functional class

Patient’s functional class was determined by the study investigator using WHO classification: [38] Class I: Patients with PH but without resulting limitation of physical activity; Class II: Patients with PH resulting in slight limitation of physical activity; Class III: Patients with PH resulting in marked limitation in physical activity; Class IV: Patients with PH with inability to carry out any physical activity.

### EuroQol-5D (EQ-5D)

The EQ-5D is a standardized, self-report measure of health status. Patients describe their health state within the domains of “Mobility”, “Self-Care”, “Pain/Discomfort” and “Anxiety/Depression” on a 3 level scales, with 1 reflecting the better health state, and rate their overall health status on a visual analogue scale (VAS) of 0 'Worst imaginable health state’ to 100 'Best imaginable health state’ [39]. The test-retest reliability of the EQ-5D has been shown to be acceptable (ICC>0.7) across a range of disease areas [40], the clinical validity of the EQ-5D has been confirmed in patients in a variety of disorders.

### Analysis

Demographic, clinical, and functional data were summarized using means and standard deviations, medians and ranges, or proportions where appropriate. Table 1 presents a summary of the analyses performed as part of the validation of the LPH. Test-retest reliability could not be analysed as part of this study given the clinical trial context and the fact that most patients experienced a change in their condition due to receiving treatment. Thus there were limited numbers of stable patient data with which to perform test-retest reliability analysis. All data processing and analyses were performed with SAS software for Windows version 9.2 (SAS Institute, Cary, NC, USA).

**Table 1:** LPH Validation analyses

**Analysis performed**	**Description**
Quality of completion of the LPH	A description of the level of completion of the LPH was performed on all questionnaires received during the study.
Description of the items of the LPH	The frequency and percentage of responses for each response choice, including missing data was described for each of the LPH items.
Description of the baseline LPH population	Patient parameters including age, gender, height and weight, blood pressure level, WHO functional class, 6MWD and Borg CR10 scale score were described at baseline.
Confirmation of the structure of the LPH	Multitrait analysis was used to confirm the item groupings in the Physical and Emotional LPH dimensions. This analysis also served to test the item convergent validity criterion of the Total score. Correlation coefficients between the Total score and Physical and Emotional dimension scores were also calculated. Confirmatory Factor Analysis (CFA) was used to confirm the structure of the LPH.
Description of the LPH Scores	The distribution of the LPH scores and changes in scores between baseline and week 12 was described. The frequency and percentage of patients scoring at floor and ceiling for each LPH score was described.
Internal consistency reliability	Internal consistency refers to the extent to which individual items are consistent with each other and reflect a single underlying construct. Cronbach’s alpha statistic is commonly used to assess internal consistency reliability. A Cronbach’s alpha coefficient of >0.70 is typically used as the criterion for acceptable internal consistency reliability [41,42]. Cronbach’s alpha was calculated at baseline (V0) on the baseline LPH population and at V6 on the V6 LPH population for the total score and for the Physical and Emotional dimension scores of the LPH.
Clinical Validity	Clinical validity evaluates the extent to which the questionnaire is able to detect variability among patients with different clinical severity levels. The LPH Total and Physical and Emotional dimension scores were described according to WHO functional class, 6MWD and Borg CR 10 Scale.
Responsiveness	Responsiveness refers to the ability of a measure to detect clinically important changes [43]. The responsiveness of the LPH Total score and Physical and Emotional dimension scores was assessed after 12 weeks. Change in scores from baseline was assessed according to change in WHO functional class (improved: change to a lower WHO functional class; stable: no change in WHO functional class; worsened: change to a higher WHO functional class), 6MWD (improved: change in 6MWD > 50; stable: -50 ≤ change in 6MWD ≤ 50; worsened: change in 6MWD <-50) and Borg CR10 Scale (improved: change in Borg CR 10 Scale < 1; stable: -1 ≤ change in Borg CR 10 Scale ≤ 1; worsened: change in Borg CR 10 Scale > 1) [44].
Minimal Important Difference (MID)	Analyses to provide an estimation of MID for the LPH scores were performed [45]. Two types of methods exist to estimate the MID: distribution-based and anchor-based methods. The main distribution-based method was based on Cohen’s effect-size; the MID was calculated as 0.2 × STD_BL_[46] and as 0.5 × STD_BL_, with STD_BL_ the standard deviation of the score at V0. The SEM was also used as a distributional estimate of MID; it was calculated as, where STD_BL_ is the standard deviation at V0 and r the reliability coefficient. Within the anchor-based methods change in scores between V0 and V6 on the WHO functional class (patients were considered 'minimally improved’ of they changed to a lower functional class), 6MWD (patients were considered 'minimally improved’ if the 6MWD increased by 50m between V0 and V6) and Borg CR10 scale (patients were considered as 'minimally improved’ if their scale decreased by 1 between V0 and V6) were used as anchors [44].

## Results

### Qualitative patient interviews

Interviews were conducted with 38 PAH patients (US n=12, Germany n=14 and France n=12). Demographic and clinical characteristics were broadly comparable across country samples, although patients in France had been diagnosed for longer than patients in the US and Germany (Table 2). Although, this may mean their symptoms were more under control, it provided a greater depth of symptom experience.

**Table 2:** Key demographic and clinical characteristics of the qualitative interview study sample (N=38)

**Demographic or clinical characteristic**	**US**	**Germany**	**France**	**Total**
	**(n=12)**	**(n=14)**	**(n=12)**	**(n=38)**
**Demographic Characteristics**				
**Age (years)**				
Mean	57.7	61.9	52.6	57.6
Median	58.5	62.5	55.5	58.5
Min, Max	41, 79	31, 85	25, 80	25, 85
**Gender n (%)**				
Male	3 (25)	3 (21)	2 (16.7)	8 (21.1)
Female	9 (75)	11 (78.6)	10 (83.3)	30 (78.9)
**Highest education level n (%)**				
Primary school	0	0	3 (25)	3 (7.9)
Before high school	0	0	1 (8.3)	1 (2.6)
Some high school	0	0	0 (0)	0 (0)
High school diploma or GED	4 (33.3)	2 (14.3)	4 (33.3)	10 (26.3)
Some years of college	1 (8.3)	0	1 (8.3)	2 (5.3)
Certificate program	0	4 (28.6)	0 (0)	4 (10.5)
College or university degree (2 or 4 year)	5 (41.6)	0	2 (16.7)	7 (18.4)
Graduate or professional degree	2 (16.7)	7 (50)	0 (0)	9 (23.7)
No education*	0	0	1 (8.3)	1 (2.6)
Skill training	0	1 (7)	0	1 (2.6)
**Clinical Characteristics**				
**Time since patient first diagnosed with PAH? (years)**				
Mean	2.2	2.7	9.4	4.3
Median	1.5	2	6.5	2.5
Min, max	0, 6	0, 8	2, 29	0, 29
**Duration of current PAH treatment? (months)**				
Mean	15.8	37.9	42.3	31.7
Median	12	23	36	21.5
Min, max	1, 48	2, 95	0, 168	0, 168
Missing data	1	0	1	2

*Education level reflects patient response that they received 'no education’.

The symptoms and domains of impact reported by patients during the open-ended part of the interviews were mapped onto the LPH items to assess content validity of the LPH. Table 3 presents the symptoms reported by five or more patients during the interviews and links them to items in the LPH. The results indicate that the key symptoms of PAH are captured by the LPH, supporting the face and content validity of the LPH with respect to the measurement of PAH symptoms. A number of other symptoms were reported by fewer than five patients each that are not assessed by the LPH. The majority of these symptoms are considered 'signs’ rather than symptoms of PAH and therefore would not be appropriate to assess.

**Table 3:** Linking patient descriptions of PAH symptoms to LPH items

Symptom*	**LPH item(s) assessing concept**
Shortness of breath (n=32)	12. making you short of breath?
Difficulty breathing (n=15)	
Tiredness (n=34)	13. making you tired, fatigued or lacking energy?
Fatigue (n=16)	
Exhaustion (n=14)	2. making you sit or lie down to rest during the day?
Weak (n=7)	
Swelling in the ankles or legs (edema) (n=31)	1. causing swelling in your ankles, legs?
Dizziness (n=13)	No item to assess this symptom
Fainting spells (n=7)	
Palpitations (n=6)	No item to assess this symptom
Headache (n=9)	No item to assess this symptom
Problems with limbs (n=7)	No item to assess this symptom

* Symptoms reported by 5 or more patients.

Table 4 presents the impacts reported by patients during the interviews and links them to items on the LPH. Patient interviews confirmed that the key impacts of PAH (those reported by five or more patients) are assessed by the LPH. For most impact concepts, there was sufficient coverage in the LPH; however, within the cognitive and emotional impact concepts there were some impacts reported that are not directly assessed. Within the cognitive concept, memory (n=26), concentration (n=24), focus (n=6) and motivation (n=5) were reported. Concentration and memory are assessed by the LPH. Focus is not directly assessed, although during the interviews patients described focus to be similar to concentration and therefore thus may be covered by the LPH in this patient population. Motivation, which was only reported by five of the 38 patients, is not assessed. A large number of emotional impacts were reported, the key impacts of worry (n=32), depression (n=28) and worry about the future (n=18) are assessed. Given these findings it can be concluded that the key impacts to patients with PAH are captured by the LPH thus supporting its content validity.

**Table 4:** Linking patient descriptions of the impact of PAH to LPH items

Impact*	**LPH item(s) assessing concept**
**Activities of daily living**	
Jobs around the home (n=36)	4. making it difficult to work around the house or in the garden?
Gardening (n=29)	5. making it difficult to go anywhere away from home?
Personal care (n=21)	
Doing shopping (n=15)	
Driving (n=11)	
Caring for children (n=6)	
Laundry (n=6)	
Vacuuming (n=6)	
Looking after animals (n=5)	
**Cognitive impacts**	
Memory (n=26)	20. making it difficult for you to concentrate or remember things?
Concentration (n=24)	
Focus (n=6)	
Motivation (n=5)	
**Emotional impacts**	
Worry (n=32)	19. making you worry?
Depression (n=28)	21. making you feel depressed?
Worried about future of PAH (n=18)	
Upset (n=9)	
Angry (n=8)	
Fear (n=8)	
Feeling low (n=7)	
Stress (n=6)	
Frustration (n=5)	
**Physical impact**	
Climbing stairs (n=37)	2. making you sit or lie down to rest during the day?
Walking (n=36)	3. making it difficult to walk about or climb stairs?
Exercise/sports (n=34)	4. making it difficult to work around the house or in the garden?
Leisure activities (n=33)	5. making it difficult to go anywhere away from home?
Carrying things (n=23)	7. making it difficult to have relationships or do things with your friends or family?
Lifting (n=17)	9. making your recreational pastimes, sports or hobbies difficult?
Bending (n=8)	
Physical movement (n=6)	
Pregnancy (n=5)	
Control of own life (n=26)	18. making you feel a loss of self-control in your life?
**Impact on relationships**	
Relationships with friends and family (n=29)	7. making it difficult to have relationships or do things with your friends or family?
Sexual activities (n=26)	10. making your sexual activities difficult?
Doing things with friends and family (n=21)	17. making you feel you are a burden to your family or friends?
**Having to rest during the day**	
Rest during the day (n=35)	2. making you sit or lie down to rest during the day?
Rest during activities (n=20)	
Rest after activities (n=10)	
**Impact on sleep**	
Problems with sleep (n=22)	2. making you sit or lie down to rest during the day?
Problems getting to sleep (n=11)	6. making it difficult to sleep well at night?
Quality of sleep (n=9)	
Problems staying asleep (n=8)	
Position of sleep (n=5)	
**Social impact**	
Social isolation (n=11)	7. making it difficult to have relationships or do things with your friends or family?
Lack of support and recognition of symptoms by friends and relatives (n=11)	17. making you feel you are a burden to your family or friends?
Socializing (n=7)	
General social impact (n=6)	
**Treatment impact**	16. giving you side effects from treatments?
Treatments intrusive and burdensome (n=10)	15. costing you money for medical care?
Work impact (n=28)	8. making it difficult to work to earn a living?
**Positive impacts**	
Support from friends and family (n=14)	7. making it difficult to have relationships or do things with your friends or family?
Impact on eating/diet (n=31)	11. making you eat less of the things you like?
**Impact on going out**	
Going out of the house (n=25)	5. making it difficult to go anywhere away from home?
Travel (n=18)	
Preventing from going out (n=10)	
Going on vacation (n=7)	
**Impact on others**	
Burden to others (n=20)	7. making it difficult to have relationships or do things with your friends or family?
Dependence on others (n=10)	17. making you feel you are a burden to your family or friends?
Impact on husband or wife (n=9)	
Needing support (n=9)	
Impact on family (n=6)	
**Financial impact**	15. costing you money for medical care?
High cost of medication (n=20)	

* Impacts reported by 5 or more patients.

The second part of the patient interviews involved cognitive debriefing of the LPH to confirm the level of understanding and relevance of the questionnaire to PAH patients. The majority of patients found the questionnaire resonated with their experience of PAH. In terms of the response options, the majority of the patients felt that the range of response options was appropriate. Some patients suggested adapting the response options or making them more specific to certain questions. While these suggestions could be taken into consideration for future versions of the LPH, the issues raised did not detract from patients’ ability to actually complete the questionnaire. Some areas to improve understanding were raised; these included highlighting the instructions to the patient to ensure they do not miss them, adapting the response options to ease completion and splitting items assessing multiple concepts into separate items. Not all patients appeared to use the recall period of one week when completing the items. The interview setting may have been one cause for this, because patients did not have a clear timeframe in which to consider their symptoms. A number of patients specifically commented that they liked the questionnaire and thought it captured appropriate symptoms. Sixteen of the patients specifically reported that they found the questionnaire 'easy’ or 'simple’ to complete.

### Psychometric validation study

The total psychometric validation population (patients who returned an LPH questionnaire at V0 or V6) included 196 patients, 190 of whom were included in the baseline population (patients who completed at least 90% of LPH items at V0), and were included in all analyses conducted on baseline data. The week 12 population (patients who completed at least 90% LPH items at V6) included 176 patients who were included in all analyses conducted on week 12 data. The responsiveness population (patients who completed at least 90% of LPH items at V0 and V6) included 171 patients who were included in responsiveness analyses. Table 5 presents patient demographic and clinical characteristics for the baseline LPH population.

**Table 5:** Description of the baseline LPH population (N=190)

**Variable **^**[a]**^	**Baseline LPH population (N=190)**
**Age**	
n (missing)	190 (0)
Mean (SD)	48.1 (16.3)
Median	48.0
Min - Max	18.0 - 80.0
**Gender**	
Male	44 (23.2%)
Female	146 (76.8%)
Missing	0 (0.0%)
**WHO functional class**	
Class I	7 (3.7%)
Class II	79 (41.6%)
Class III	101 (53.2%)
Class IV	2 (1.1%)
Missing	1 (0.5%)
**6 Minute Walking Test at baseline**	
n (missing)	188 (2)
Mean (SD)	365.0 (67.7)
Median	380.0
Min - Max	160.0-468.0
**Borg Score at baseline**	
n (missing)	187 (3)
Mean (SD)	3.8 (2.1)
Median	3.0
Min - Max	0.0-10.0

[a] Missing data included in calculation of percentages.

### Quality of completion of the LPH

Over 85% patients had no missing items on the LPH at both baseline and week 12. All LPH items had less than 3% missing data at baseline and week 12. Responses were well spread across the response scale. The results indicate a good level of completion for the LPH items and questionnaires consistent with the benefits of a single page instrument and reports from the qualitative work that the questionnaire was easy to complete.

### Scaling properties and confirmation of the structure of the LPH

Multitrait analysis performed on the LPH scale scores and total score at baseline and visit 6/last visit indicated that for both the Emotional and Physical scores all items met the criteria for item convergent and item discriminant validity. Item-scale correlations ranged from 0.59-0.76 for the 'Emotional’ score and 0.43-0.78 for the 'Physical’ score. For the Total score all but two items met the test for item convergent validity (correlation range: 0.38-0.72). Two items correlated with the Total score at a level of r=0.38 which is just below the threshold of acceptability (r=0.40). There was a moderate correlation between the LPH Emotional and Physical scores (r=0.58) indicating that the scales are related but not redundant and high correlations between the LPH Total Score and both the LPH Emotional score (r=0.85) and the LPH Physical score (r=0.87), which indicates that the Total Score adequately covers both physical and emotional dimensions. At both baseline and week 12 the percentages of patients with the lowest or highest possible score was low (<3%) for all scales, indicating no floor or ceiling effect for the LPH scores. These results indicate a good ability for patients to both improve and worsen on the scales (Table 6). Finally, strong internal consistency reliability of the LPH scores was demonstrated by Cronbach’s alpha values of >0.70 for all LPH scores at baseline and week 12 (Table 6).

**Table 6:** Summary of LPH Properties

**Dimension**	**Structural properties of the LPH**				**Percentages of patients having the lowest or highest possible LPH scores**						**Internal consistency reliability of LPH scores**			
	**# of items**	**Range of item-scale correlations**	**% of items meeting convergent validity criterion**^**1**^	**% of items meeting discriminant validity criterion**^**2**^	**Lowest possible score**	**Highest possible score**	**Baseline LPH population (N=190)**		**Visit 6/last visit LPH population (N=176)**		**Baseline**		**Visit 6/last visit**	
							**N (%) at floor**	**N (%) at ceiling**	**N (%) at floor**	**N (%) at ceiling**	**N**	**Cronbach’s Alpha**	**N**	**Cronbach’s Alpha**
LPH Emotional score	5	0.59-0.76	100%	100%	0	25	16 (8.42%)	6 (3.16%)	16 (9.09%)	2 (1.14%)	189	0.87	176	0.87
LPH Physical score	8	0.43-0.78	100%	100%	0	40	4 (2.11%)	0 (0.00%)	0 (0.00%)	0 (0.00%)	190	0.89	175	0.90
LPH Total score	21	0.38-0.72	90%	100%	0	105	2 (1.05%)	0 (0.00%)	0 (0.00%)	0 (0.00%)	185	0.92	171	0.92

^1^ Correlation between each item and its own scale corrected for overlap should be at least 0.40.

^2^ Each item should correlated significantly higher with its hypothesized scale than with the other scale in the measure.

Confirmatory factor analysis (CFA) of the LPH Emotional and Physical scores at baseline indicated an average overall fit for the items on the Emotional and Physical scores. CFA of the LPH Total score at baseline indicated a poor overall fit of the model with poor factor loadings for most LPH items with only five items meeting the criteria of 0.70.

### Clinical validity

**Figure 2:**
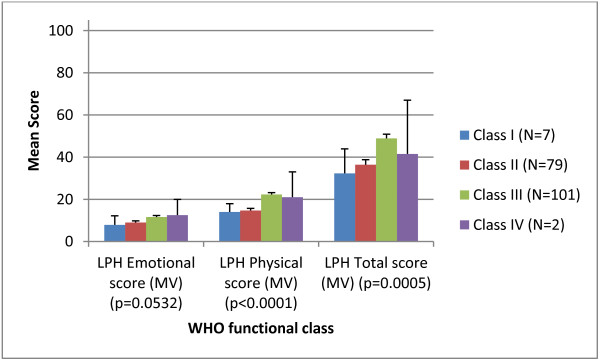
Clinical validity of the LPH scores at baseline according to the WHO functional class (N=189).

**Figure 3:**
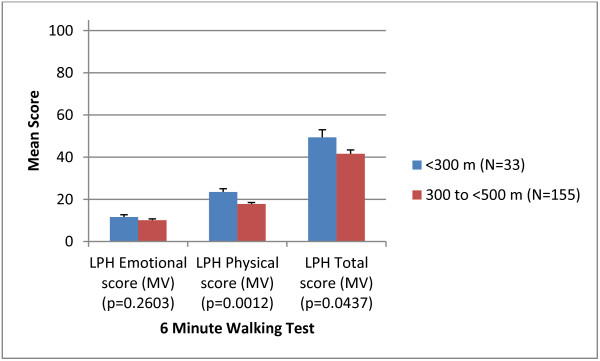
Clinical validity of the LPH scores at baseline according to the 6 Minute Walking Test (N=188).

Results indicated that the LPH Physical and Total scores were able to discriminate among groups of patients of differing severity levels, as defined by World Health Organisation (WHO) functional class (Figure 2) and the six minute walking test (Figure 3). The LPH Emotional score did not discriminate between severity groups at a statistically significant level. Small sample sizes in WHO class I and IV should be considered when interpreting these results. However, the LPH Emotional, Physical and Total scores were broadly worse for those subjects with more severe disease across clinical criteria.

Correlations were examined between LPH scores with the Borg score at baseline and V6 (week 12). The highest Borg correlations were with the LPH Physical score, as expected (r=0.36 and r=0.34 respectively). Correlations with the LPH Emotional Score (r=0.11 and r=0.15), and the LPH Total Score (r=0.21 and r=0.23) were low.

### Concurrent validity

The pattern of correlations between the LPH scores and the concurrent measures was consistent with the content of the different scales, and so supportive of the validity of the measure. Scales measuring similar concepts correlated more highly than scales measuring dissimilar concepts (Table 7). For example, the LPH Emotional score correlated moderately with the EQ-5D anxiety/depression item (0.59), but at a low level with EQ-5D self-care (0.24).

**Table 7:** Validity of the LPH scores

	**LPH score**			**LPH score**		
	**LPH emotional score**	**LPH physical score**	**LPH total score**	**LPH emotional score**	**LPH physical score**	**LPH total score**
**Correlations with clinical parameters**	**Baseline**			**Week 12**		
Borg score	0.11	0.36	0.21	0.15	0.34	0.23
**Concurrent validity with EQ-5D items**	**Baseline (N=190)**			**Week 12**		
Mobility	0.26	**0.51**	**0.41**	0.12	0.37	0.27
Self-Care	0.24	0.32	0.35	0.25	**0.44**	**0.40**
Usual Activities	0.36	**0.46**	**0.45**	0.30	**0.48**	**0.41**
Pain/Discomfort	0.35	0.36	**0.40**	0.23	0.28	0.27
Anxiety/Depression	**0.59**	**0.42**	**0.53**	**0.51**	0.34	**0.41**
EQ-5D VAS	0.26*	**0.48**	0.35	-0.20	**-0.52**	-0.36
EQ-5D index	**0.49**	**0.53**	**0.57**	**-0.42**	**-0.52**	**-0.48**

Bolded numbers = Statistically significant result.

*N=189.

Table 8 presents a summary of the responsiveness analyses for the LPH. The results provide some support for the responsiveness of the Physical score and the Total score, but not the Emotional score. For the Physical score and the Total score, across all three methods of defining change groups, effect sizes suggest there were small to moderate improvements for the 'improved’ group, small improvements in the 'stable’ group and negligible change in the 'worsened’ group. However, for the Emotional score there were only small improvements in both the 'improved’ and 'stable’ groups, and negligible change in the worsened group. Moreover, the differences between change groups was only significant for the LPH Physical and Total Score according to change in Borg score (p=0.0073, p=0.0415 respectively).

**Table 8:** Responsiveness of the LPH

**Responsiveness criteria**	**Value**	**LPH emotional score**				**LPH physical score**				**LPH total score**			
		**Improved**	**Stable**	**Worsened**	**p-value**	**Improved**	**Stable**	**Worsened**	**p-value**	**Improved**	**Stable**	**Worsened**	**p-value**
WHO Functional Class	N	37	123	10	p=0.9791	38	123	10	p=0.0976	37	123	10	p=0.5096
	Mean Change	-1.5	-1.6	-1.3		-4.2	-2.3	1.6		-6.3	-6.1	1.0	
	Effect Size	-0.20	-0.22	-0.14		-0.54	-0.23	-0.19		-0.32	-0.27	0.04	
Six Minute Walking test	N	51	110	10	p=0.7050	51	110	10	p=0.1446	51	110	10	p=0.6578
	Mean Change	-1.7	-1.6	0.1		-3.3	-2.3	0.6		-6.7	-5.3	-3.4	
	Effect Size	-0.22	-0.21	0.01		-0.35	-0.25	0.07		-0.29	-0.24	-0.16	
Borg score	N	36	121	13	p=0.2237	36	121	13	p=0.0073	36	121	13	p=0.0415
	Mean Change	-1.7	-1.6	0.5		-5.3	-1.9	0.2		-9.9	-4.9	0.3	
	Effect Size	-0.26	-0.21	0.06		-0.61	-0.20	0.02		-0.46	-0.23	0.01	

Note: For the Mean Change a negative figure denotes improvement in score.

### Interpretation of the LPH Scores

Results to estimate the MID for the LPH scores using anchor-based methods indicate an MID with a range of 1.48-3.69 for the LPH Emotional Score, 1.88 to 4.71 for the LPH Physical Score and 4.41 to 11.02 for the LPH Total score (Table 9).

**Table 9:** Estimation of MID for the LPH from baseline to visit 6/last visit

**LPH scores**	**N**	**Standard deviation at baseline**	**MID using ES=0.2**	**MID using ES=0.5**	**MID using SEM**
LPH Emotional score	190	7.38	1.48	3.69	2.66
LPH Physical score	190	9.41	1.88	4.71	3.12
LPH Total score	190	22.03	4.41	11.02	6.23

## Discussion

The results of this study provide evidence that the LPH has strong content validity and psychometric validity as a measure of symptoms and HRQoL in PAH. Mapping the symptoms and domains of impact elicited from the patient interviews on to the LPH items indicated that key symptom concepts relevant to PAH are assessed by the LPH. A number of other symptoms were reported by patients that are not directly assessed by the LPH, but none of them were felt to be primary symptoms. Moreover, although a wide range of additional impacts were also mentioned, they were very personal and reflected the individual’s living situation rather than their PAH experience and so would be unlikely to resonate with the wider PAH population. While patients discussed a number of emotional impacts that are not specifically addressed by the LPH, including items to assess all of the emotional impacts would add significant respondent burden. Furthermore, some of the concepts such as 'upset’ and 'feeling low’ are vague concepts, and ones which are to some extent covered by the items that are included in the LPH (e.g. feeling depressed). Considering patient burden and relevance, the instrument captures all core symptom and impact domains.

In addition to the concept elicitation results, when the LPH was debriefed with patients it was well understood and the questions were considered relevant, with no concerns raised that key concepts were missing, thus providing further evidence of content validity. The LPH response options were understood well as evidenced by the high proportion of patients who were able to rate the symptoms and impact of PAH on different aspects of their life as defined in the questionnaire. However, some patients suggested further response descriptors in addition to the existing 'No’, 'Very little’ and 'Very much’ descriptors. While additional descriptors might be useful in future versions of the LPH, their absence was unlikely to have affected the ability of patients to complete the current version of the LPH. Moreover, there is evidence that increasing the number of categories does not always provide a larger coverage of the target trait, and they concluded that rating scales with fewer response categories were more functional [47]. Although many patients found the 'past week’ recall appropriate, some patients felt this was too short a time period over which to consider the impact of PAH on their lives. The FDA suggests the use of shorter recall periods over longer periods; [13] patients themselves note it would be harder for them to accurately recall their symptoms over a longer period of time. Thus, extending the recall period would not be appropriate.

The results of scaling tests supported the a priori structure of the LPH. However, as scale development is an iterative process, some modifications to the LPH may be warranted to improve the structure further for future studies. Nevertheless, a trade-off between the advantages and disadvantages of changing a well-validated and now-well used scale with interpretation aids should be considered before making any modifications. The results of a confirmatory factor analysis on the LPH Emotional and Physical scales were positive, with goodness of fit values indicating an average overall fit and moderate factor loadings on the factor analysis model. Further testing using exploratory factor analysis could be considered to explore whether an alternative structure of the LPH would produce a better fitting model. However, as noted above there is a trade-off to be considered when making modifications to a well-validated and well-used instrument. Internal consistency reliability results were very good for the LPH sub-scale scores and the Total score indicating that the LPH items included on each scale are measuring a single underlying concept, without being redundant. These results are consistent with those demonstrated by Rector and colleagues in the validation of the original MLHFQ in heart failure patients [48]. It was not possible to evaluate test-retest reliability as part of this study – such testing is recommended as a priority in future evaluations of the LPH.

Clinical validity was evaluated by examining the ability of the instrument to discriminate between patients who differed on key clinical indicators. The results provided good evidence of clinical or known groups validity for the Physical score and the Total score (with statistically significant differences among groups), but limited support for the clinical validity of the Emotional score. While the pattern of mean scores for the emotional score was in line with expectations, the differences between groups were not statistically significant. However, low sample sizes for these analyses, particularly in the case of WHO functional class categories I and IV should be considered when interpreting these results. In addition, the emotional subscale contains fewer items than the physical subscale (5 vs. 8) and therefore differences in scores between clinical groups may not be as apparent. Concurrent validity tests demonstrated that in most cases there were moderate correlations between the LPH scores and EQ-5D items that would be expected to correlate. These results indicate that the two questionnaires are measuring similar concepts but are not redundant with each other and are thus supportive of the validity of the LPH. The clinical and concurrent validity properties were comparable to those for similar scales on the CAMPHOR, a PAH specific instrument [12].

The responsiveness results provided mixed support regarding the ability of the LPH to reflect changes over time, and suggest that the Physical score and the Total score are more responsive to change over time than the Emotional score. However, this is to be expected within the context of a short term trial, as emotional functioning is typically more distal than physical functioning, and is often less responsive to treatment. These findings are consistent with those presented by Gilbert et al. in reporting the MID for the 6MWD and Short Form-36 (SF-36); they similarly found that significant improvements were only demonstrated for the 6MWD and SF-36 physical functioning scale [9]. Overall, the responsiveness of the LPH can be concluded to be acceptable. However, further investigations of responsiveness are arguably warranted, particularly to assess responsiveness to worsening and the responsiveness characteristics of the Emotional scale within the context of a larger scale study.

Analyses to provide guidance for interpretation of LPH scores using distribution-based methods indicated that for all LPH scores there was a small range of MID definition. Given this, based on an average of the results, a recommendation for a change of three points on the sub-scales, and a change of seven points on the Total score would be considered as meeting the MID. This would translate as a patient who experienced improvement in only a few areas of their condition perceiving that they had experienced a beneficial improvement in their quality of life. It is likely that the 'important’ or clinically important difference is higher –around 4 points for the sub-scales and 11 points for the Total score, as a 0.5 ES is easier to argue, given the literature available to back it up [49,50]. Estimation of MID using anchor-based methods could not be established in this study due to correlations in LPH scores and change in clinical parameters indicating no clear linear relationship. Therefore, further investigation of MID using anchor-based methods is recommended.

This study provide evidence supporting the use of the LPH over other PAH specific instruments such as the CAMPHOR. The original MLHFQ and now the LPH have a strong history of published qualitative research supporting content validity and use in clinical trials. The evidence of these factors for the CAMPHOR is limited. Although the CAMPHOR was developed specifically for a PAH population, the development and validation samples were very homogenous, including only white English speaking patients, thus limiting the generalizability of the instrument [12]. The LPH however, has been validated using a large sample of PAH patients across a range of cultures and languages. The CAMPHOR authors also acknowledge that a limitation of their instrument is lack of evidence of responsiveness to treatment within a clinical trial [12]. This paper provides evidence for the responsiveness of the LPH in an interventional study with PAH patients. Finally, the LPH can be considered a more suitable instrument for use with PAH patients given its short length (21 items), compared to the longer CAMPHOR (65 items) and the fact it has clear, PAH-specific instructions, something the CAMPHOR lacks.

### Limitations

Although this study provides strong support for the face and content validity, and measurement properties of the LPH there are some limitations that should be acknowledged. The cut-points for the 6MWD and Borg Scale [44] as used for the responsiveness and MID analyses were selected based on evidence from published literature. For the 6MWD at the time of SAP design there was only one published study which presented a suggested MID for the 6MWD in PAH. This study recommended an MID of 41m and a range of 18.70-74.15 [9] which the cut-point used in this study was within. However, the authors are aware that since the analyses were completed there have been cut-points published for the two instruments that could be considered more clinically relevant to distinguish groups for clinical validity testing. For future studies and further validation of the instruments such cut-points will be considered.

The authors also note that it would have been beneficial to collect haemodynamic characteristics in order to characterise the sample, but unfortunately this was not collected. However, it is still felt that the sample is well characterised and clinically relevant.

## Conclusion

Overall, this study provides evidence that the LPH has good face and content validity in patients with PAH, has strong psychometric properties and is appropriate for use in clinical trials as a measure of core symptom and impact concepts.

## Abbreviations

6MWD: Six-minute walk distance; 6MWT: Six-minute walk test; CAMPHOR: Cambridge pulmonary hypertension outcome review; CR10: Borg category ratio 10; EQ-5D: EuroQoL-5D; ERA: Endothelin receptor antagonist; HRQoL: Health related quality of life; IV: Intravenous; LPH: Living with pulmonary hypertension; MID: Minimal important difference; MLHF: Minnesota living with heart failure; PAH: Pulmonary arterial hypertension; PDE5I: Phosphodiesterase type 5 inhibitor; QoL: Quality of life; VAS: Visual analogue scale; VI: Visit 1; WHO: World health organisation.

## Competing interests

Bayer Pharma AG funded this study; Ms Bonner, Ms Abetz and Ms Meunier were contracted by Bayer Pharma AG as consultants to the study and develop the manuscript. Dr. Mathai was employed by Bayer Pharma AG as a scientific advisor. The authors declare there are no other competing interests.

## Authors’ contributions

NB and LA were involved in the conception, design and conduct of the qualitative and quantitative components of the study and the interpretation of all study findings. NB drafted the manuscript, LA reviewed the manuscript critically for scientific content. JM was involved in the design, conduct and analysis of the quantitative component of the study and reviewed the manuscript critically for content. MS was involved in the interpretation of study findings and drafting of the manuscript. SM critically reviewed the manuscript for scientific content and was involved in the revision of the manuscript. All authors read and approved the final manuscript.
